# Connectivity Study of the Neuromechanism of Acute Acupuncture Needling during fMRI in “Overweight” Subjects

**DOI:** 10.1155/2015/384389

**Published:** 2015-03-02

**Authors:** Karen M. von Deneen, Wei Qin, Peng Liu, Minghao Dong, Peng Chen, Huisheng Xie, Yi Zhang, Mark S. Gold, Yijun Liu, Jie Tian

**Affiliations:** ^1^School of Life Science and Technology, Xidian University, Xi'an 710071, China; ^2^Department of Psychiatry & McKnight Brain Institute, University of Florida, 1149 S. Newell Dr. L4-100K, Gainesville, FL 32610, USA; ^3^Beijing University of Chinese Medicine, Beijing 100029, China; ^4^Department of Small Animal Clinical Sciences, College of Veterinary Medicine, University of Florida, P.O. Box 100126 2015 SW 16th Avenue, Gainesville, FL 32610, USA

## Abstract

This functional connectivity study depicts how acupoints ST 36 and SP 9 and their sham acupoints acutely act on blood glucose (GLU), core body temperature (CBT), hunger, and sensations pertaining to needling (*De-qi*) via the limbic system and dopamine (DA) to affect various brain areas in fasting, adult, and “overweight” Chinese males using functional magnetic resonance imaging. Functional connectivity (FC) analysis utilized the amygdala (AMY) and hypothalamus (HYP) as regions of interest (ROIs) in the discrete cosine transform and seed correlation analysis methods. There was a significant difference in the spatial patterns of the distinct brain regions between groups. Correlation results showed that increased HYP-hippocampus FC after ACU was positively correlated with ACU-induced change in CBT; increased HYP-putamen-insula FC after ACU was positively correlated with ACU-induced change in GLU; and increased HYP-anterior cingulate cortex FC after ACU was positively correlated with ACU-induced change in HUNGER suggesting that increased DA modulation during ACU was probably associated with increased poststimulation limbic system and spinothalamic tract connectivity. Decreased HYP-thalamus FC after ACU was negatively correlated or anticorrelated with ACU-induced change in HUNGER suggesting that increased DA modulation during ACU was possibly associated with decreased poststimulation limbic system and spinothalamic tract connectivity. No correlation was found for min SHAM. This was an important study in addressing acute acupuncture effects and neural pathways involving physiology and appetite regulation in overweight individuals.

## 1. Introduction

Acupuncture has been reported to be an effective therapeutic tool for obesity, but its mechanisms remain unclear. The knowledge of the hormonal interplay and resulting brain activations is crucial in understanding how acupuncture specifically affects energy and feeding mechanisms in overweight and obese populations, as shown in previous obesity acupuncture studies (see recent reviews by Belivani et al. 2013 [1], Sui et al. 2012 [2]). Treating obesity with acupuncture is associated with a milieu of hormones and physiological functions. For example, Pissios and Maratos-Flier [3] proposed that central serotonin affects GLU (glucose) homeostasis since inhibition of serotonin reuptake decreases appetite. Apparently, the arcuate nucleus proopiomelanocortin (ARC POMC) neurons respond to serotonin as well as leptin and GLU, which are affected by real acupuncture (ACU) treatment [4]. Low leptin and other adipokine levels during fasting stimulate food intake and decrease basal metabolic rate (BMR) (for full review see [5]). Leptin controls GLU and lipid metabolism via AMP-activated protein kinase and stearoyl-coenzyme A desaturase 1 in the liver and muscle [6], which may be targeted by ACU treatment. Brain regions involved in satiety that may be involved in this study are the inferior parietal lobes, dorsolateral prefrontal cortex (DLPFC), and ventromedial prefrontal cortex (VMPFC) [7]. Our lab has shown that there is a delayed hypothalamic response to reach satiety in obese individuals [7, 8]; hence it would be interesting to determine if that is the case in overweight individuals.

One of our initial studies helped address areas in the brain activated by ACU that could suppress appetite and prevent weight gain by decreasing food intake. James et al. [7] showed activation in the insula (INS), prefrontal cortex (PFC), amygdala (AMY), thalamus, nucleus accumbens (nACC), and ventral basal ganglia in hungry subjects when viewing food versus nonfood related international affective picture system (IAPS) photographs. Data from reward pathways showed that images of rich, fattening food induced significantly greater activation than nonfood object pictures in the left and right striatum (ventral striatum, putamen, andcaudate), as well as the midbrain (including the ventral tegmental area), left AMY, and left orbitofrontal cortex (OFC) [9]. These pathways integrate aspects of motivation for feeding with hypothalamic inputs on the state of energy balance [10].

Most importantly, the hypothalamus (HYP) is a key component to feeding behavior; thus it is significant to look into its regulation in more detail. There have been five individual areas identified in the HYP that regulate feeding behavior and metabolism [11]. Medial areas of the HYP control food intake and energy homeostasis. These regions obtain important information from referring organs and systems that are involved in nutrient and metabolite consumption and distribution, as well as involvement in hyperphagia and obesity [12]. Ghrelin and leptin have been known to target the HYP in regulating feeding behavior. Leptin activates its receptors so that neuropeptide Y (NPY), orexin (ORX), *β*-endorphin, and alpha melanocyte-stimulating hormone (*α*-MSH) can decrease appetite stimulation [13]. Leptin has been shown to have an important role in appetite control. Leptin can suppress ghrelin expression at the level of NPY neurons [14, 15]. The roles of leptin and ghrelin feedback on the appetite regulating network (ARN) are crucial for energy homeostasis and appetite [16]. If there is a drop in leptin levels in the blood, ARN is stimulated to release orexigenic NPY, agouti related protein (AgrP), and gamma-aminobutyric acid (GABA) along with an inhibition of anorexigenic *α*-MSH [13]. This can be best summarized by Erlanson-Albertsson [17].

It is then necessary to discuss the interactive pathways that regulate appetite and cravings. ARN has appetite enhancing and reducing circuits that are located in the arcuate-paraventricular nucleus (ARC-PVN) axis of the HYP. It is affected by signaling from the lateral hypothalamus (LHA) and ventromedial hypothalamus (VMH) [13]. These particular pathways have their components synthesized in ARC and are targeted at the parvocellular PVN (pPVN) and magnocellular PVN (mPVN), which may provide insight on the mechanisms of overweight subjects. The release of these neurochemicals is regulated primarily by the VMH and LHA [11]. Kalra and colleagues [11] showed that if there was a disruption between these two sites, then the affected individual would overeat and gain weight. This suggests that the VMH is responsible for inhibiting signals to ARC. Certain areas in LHA that express ORX or melanin-concentrating hormone (MCH) increase NPY release, thereby stimulating appetite. Thus, if there is nonstop stimulation of NPY receptors, then the satiety signal to the HYP is inhibited resulting in continuous eating [11], which is a typical symptom in overweight individuals. Despite this, there is no known receptor downregulation for NPY [18]. It was shown that, during the absence or decrease of food intake, NPY levels increased in ARC in order to stimulate appetite [11].

Overall, acupuncture is one of the most important therapeutic modalities in traditional Chinese medicine (TCM) [19], especially in treating obesity [1, 2]. While acupuncture has gained popularity in the Western medical community, the underlying mechanisms remain undefined in weight loss. We expected to see activation due to acupuncture effects in the VMH/LHA, medial prefrontal cortex (mPFC), and ventral striatal regions related to glycometabolism [20]; in the HYP and brain stem affecting gastric function and the central nervous system (CNS) [21]; in the PVN, cerebral cortex, subcortical structures (AMY, hippocampus (HIPP), cerebellum, and thalamus) involving cognitive function [22]; and in the ARC, inferior parietal lobes, DLPFC/VMPFC, INS, and ventral basal ganglia engaging satiety (see review [23]). In the current study, we investigated the spatial and temporal patterns of brain responses modulated by the effects of manual acupuncture at ST 36 (*Zu San Li*) and SP 9 (*Yin Ling Quan*), employing the discrete cosine basis set (DCT) [24], Pearson correlation (http://www.statisticshowto.com/what-is-the-pearson-correlation-coefficient/), and functional connectivity methods [25–27].

## 2. Materials and Methods

### 2.1. Subjects

The study was performed on 19 right-handed volunteer Chinese males aged 21–45 years (10 for ACU treatment and 9 for minimal sham acupuncture treatment (min SHAM)) with a body mass index (BMI) >18 and <30 and who had no history of major neurologic and psychiatric disease. Other exclusion criteria included having an athletic or fit physique, waist-to-hip ratio (WHR) <0.9, smokers, being on a weight-loss program, claustrophobic, not abstaining from eating 12 hrs prior to scanning, and being on antidepressants and/or appetite suppressing medications. All subjects were acupuncture naïve and gave written informed consent as approved by the West China University of Medical Science. The experiments were carried out in accordance with the Declaration of Helsinki. All patients were free to withdraw from the study at any time without obligation.

### 2.2. Experimental Design

**Figure 1:**
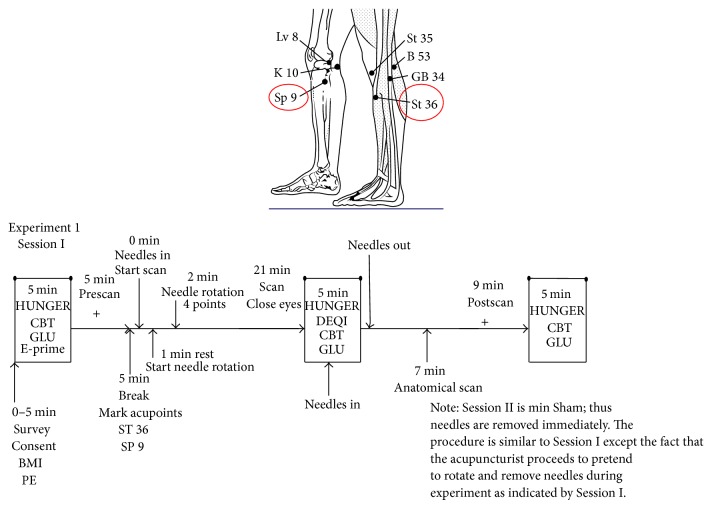
Description of functional magnetic resonance imaging experimental design for acupuncture done on right-handed, overweight Chinese males (age 21–45 years). Diagram shows the location of the two acupoints, ST 36 and SP 9, used for the acupuncture experiment. Experiment  1 Session I delineates the progression of the experimental procedures for *n* = 10 patients. Experiment  2 Session II for the minimal Sham procedure (*n* = 9) is described verbally in the note. Abbreviations: CBT: core body temperature; GLU: glucose; BMI: body mass index; PE: physical examination; http://bhojraj.tripod.com/July03knee.gif.

Subjects were recruited and prescreened based on a standard questionnaire. Subjects were randomly assigned by a computer program to groups A and B (the acupuncturist was the only nonblinded individual in the research group). Group A received the standard ACU treatment. Group B was treated with the min SHAM treatment. Experiment  1 Session I (which included group A) and Experiment  2 Session II (which included group B) consisted of the following protocols. For a thorough explanation of the experimental setup, please refer to Figure 1.

### 2.3. Physiological Measurements

Height (cm) and weight (kg) were measured for each subject in order to calculate the BMI, as well as the WHR. A brief chest and heart auscultation was performed on each patient. Prior to scanning, initial core body temperature (CBT) was measured sublingually with an Omron electronic thermometer (MC-142L). Initial GLU was taken from the left index finger and was measured via the OneTouch Ultra Blood Glucose Monitoring System (Lifescan; Johnson & Johnson Company). The instrument used glucose oxide (>0.8 IU) and a buffer (0.05 mg). Its range was 20–600 mg/dL or 1.1–33.3 mmol/L. Accuracy was a slope of 0.986,* y*-intercept = −5.5 mg/dL, and CC = 0.984. Precision was 1.6–3.2% for blood and 2.4–4.4% for the control. Blood pressure was measured via the Omron electronic blood pressure monitor (HEM-645). Sensitivity was ±4 mmHg (±5% accuracy) with a range of 0–299 mmHg. The hunger survey was then conducted asking the patient to evaluate his hunger on a standard Likert scale from 0 (no hunger) to 10 (starvation).

After a 21 min scan, during which the ACU or min SHAM treatment was done (described below and in Figure 1), CBT, GLU (taken from the right index finger), and a hunger survey were conducted. The patient was asked to evaluate the* De-qi* sensations he felt during the treatment. A standard Likert scale (0 being no sensation and 10 being the most intense sensation felt) was used to evaluate the* De-qi* sensations listed. When the anatomic scan and postscan were done, the final CBT, GLU (left middle finger), and a hunger survey were conducted. Subjects were asked if they thought they received real or sham acupuncture.

### 2.4. Treatment Methods

After a 5 min prescan, the certified acupuncturist set up for either the ACU or min SHAM procedure, depending on random patient assignment. The scan began when needles were inserted at time 0 min. For ACU, 4 acupoints were used bilaterally, ST 36 and SP 9. ST 36 is 3 cun below ST 35 (*Du Bi*), which is in the depression lateral to the patellar ligament on the lower border of the patella when the knee is flexed and 1 cun lateral to the anterior crest of the tibia. When the knee is flexed, SP 9 is located along the posterior border of the upper tibia. For min SHAM, the SHAM acupoints were located 2 cun lateral and dorsal to ST 36 and 2 cun medial to SP 9 on the same plane bilaterally. The acupuncturist used paramagnetic (0.18 mm × 40 mm) needles for both ACU and min SHAM.

For ACU, after a 1 min pause, the acupuncturist inserted needles vertically to a depth of 2-3 cm and rotated needles in a “tonifying and reducing” technique clockwise and counterclockwise at a rate of 60 times per minute (2 Hz) in an alternating bilateral diagonal manner at 30 sec intervals for a total of 2 min. The subject was allowed to raise his right index finger if the* De-qi* sensations were painful. The lower legs were covered to mask the treatment choice. The scan continued for 21 min with the needles in.

For min SHAM, after a 1 min pause, the acupuncturist inserted the needles superficially and immediately removed them but pretended to rotate the needles as described for the ACU procedure. The lower legs were covered to mask the treatment choice. After either treatment, a 7 min anatomical scan and a 9 min postscan were conducted.

### 2.5. Imaging

The functional magnetic resonance imaging (fMRI) experiment was performed using a 3.0 Tesla Signa (GE) MRI scanner with a standard head coil. The images covered the entire brain and were parallel to the AC-PC line. Functional images were acquired with a single-shot gradient–recalled echo planar imaging (EPI) sequence (TR/TE: 2000 ms/30 ms, field of view (FOV): 240 mm × 240 mm, matrix size: 64 × 64, flip angle: 90°, in-plane resolution: 3.75 mm × 3.75 mm, and slice thickness: 5 mm thick with no gaps, 43 sagittal slices). A set of T1-weighted high-resolution structural images was collected (TR/TE: 5.7 ms/2.2 ms, FOV: 256 mm × 256 mm, matrix size: 256 × 256, flip angle: 7°, in-plane resolution: 1 mm × 1 mm, and slice thickness: 1 mm with no gaps).

### 2.6. Preprocessing of Data and Analysis

The first 5 time points were discarded to avoid the instability of the initial MRI signal. Data sets were preprocessed using statistical parametric mapping 5 (SPM5) (http://www.fil.ion.ucl.ac.uk/spm/). Images were realigned to the first image. If translation and rotation was >1 mm in any direction or >1 degree, the subject was excluded. The images were then normalized to a Montreal Neurological Institute (MNI) template and resampled to 3 mm × 3 mm × 3 mm.

The first 0.5 min of data was omitted and 8.5 min of the ACU normalized data were extracted. The data were then smoothed with a 12 mm full-width at half maximum (FWHM) Gaussian kernel for the discrete cosine transform (DCT) analysis. DCT is effective in detecting spatial patterns of any signal change during a specific band frequency [24]. DCT analysis was followed by steps depicted in Liu et al. [19]. The discrete cosine bias set contained 60 regressors spanning the frequency of 0–0.1 Hz. Statistical parametrical maps were constructed by computing* F*-contrasts, which compared the effect of signal fluctuations in the range of 0.01–0.1 Hz. Statistical parametrical maps were created under the threshold *P* < 0.005 (corrected for multiple comparisons) at the first level. The final overlapping mask was created by multiplying the binary values of the individual mask in each group. Finally, the conjunction analysis of the two group masks was applied to detect intergroup similarities of spatial patterns, which was adopted as the region of interest (ROI) for the functional connectivity analysis. Functional connectivity describes the temporal synchrony or correlation of the blood oxygen level-dependent (BOLD) signal during functional imaging from two or more anatomically separate brain regions [25] by detecting coherence in fMRI signals among these regions during either a behavioral task or a resting state engaging no task. Such a functional reference is often determined by an ROI in a brain activation study using specific behavioral tasks or external stimuli [26].

Therefore, by using Talairach coordinates, SPM5, and DCT analysis, the overlapped regions were the HYP and AMY, and they were chosen as ROIs for the functional connectivity results for the whole brain. For each subject, the seed correlation analysis (SCA) was conducted between the seed reference and the rest of the whole brain in a voxel-wise manner by regressing out the effects of head motion parameters and signals from a region centered in the white matter and a region centered in the cerebrospinal fluid. For each subject, correlation coefficients were then converted to an approximately normal distribution using Fisher's* z*-transformation. At the second-level analysis, a two sample *t*-test was applied to evaluate the baseline scan of the two acupoint groups before ACU or min SHAM. Finally, the test for differences of the brain networks between the two groups was evaluated using a two sample *t*-test. All contrasts had a threshold at *P* < 0.005 (uncorrected) and a cluster size >3 voxels.

## 3. Results and Discussion

**Figure 2:**
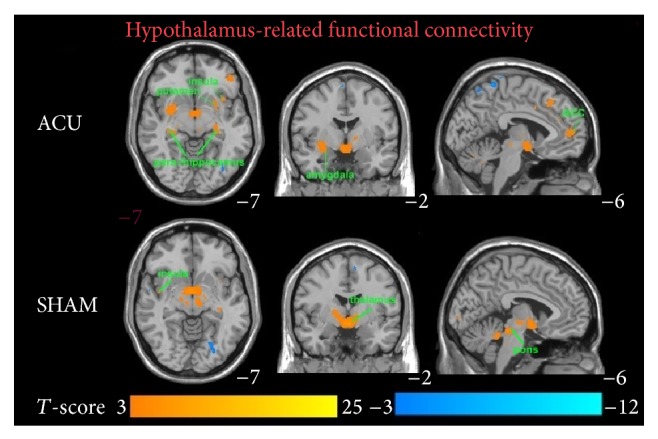
Blood oxygen level-dependent (BOLD) signals in significant brain regions from a hypothalamus-related functional connectivity analysis comparing real acupuncture (ACU) versus minimal sham- (min SHAM-) treated individuals (*n* = 10 and *n* = 9, resp.). Epoch of treatment lasted 9 min. Results from the conjunction analysis were based on the discrete cosine transform (DCT) group results for acupoints ST 36 and SP 9. The overlapping areas are the putamen, insula, parahippocampus, hippocampus, amygdala, anterior cingulate cortex (ACC), thalamus, and pons. *T*-value scales are located on the bottom of the picture (*P* < 0.05).

**Figure 3:**
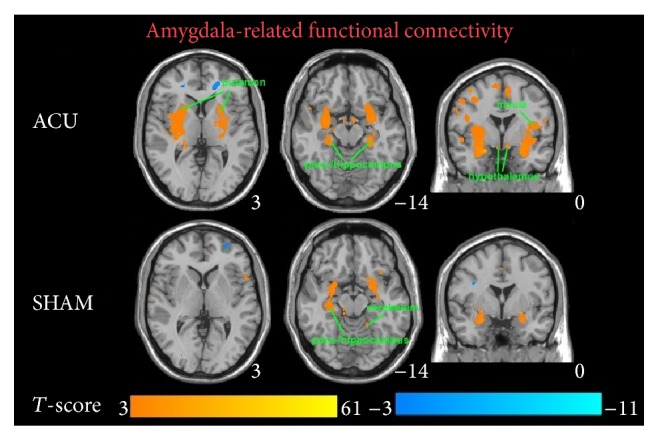
Blood oxygen level-dependent (BOLD) signals in significant brain regions from an amygdala-related functional connectivity analysis comparing real acupuncture (ACU) versus minimal sham- (min SHAM-) treated individuals (*n* = 10 and *n* = 9, resp.). Epoch of treatment lasted 9 min. Results from the conjunction analysis were based on the discrete cosine transform (DCT) group results for acupoints ST 36 and SP 9. The overlapping areas are the putamen, insula, parahippocampus, hippocampus, hypothalamus, and cerebellum. *T*-value scales are located on the bottom of the picture (*P* < 0.05).

SCA results comparing activations between ACU and min SHAM and their overlapping areas from the functional connectivity analyses are listed in Tables 1 to 4, respectively. As shown, there are significant differences between HYP (see Figure 2) versus the left and right AMY (see Figure 3) as well as between ACU versus min SHAM. Brain regions comparing activations between ACU versus min SHAM are shown in Table 1. ROI used for the comparisons was the HYP. This was an uncorrected comparison set at *P* = 0.005 with a *T* value of 2.898. The key areas for ACU were the superior temporal gyrus, middle occipital gyrus, vermis 3, temporal pole: superior temporal gyrus, HIPP, fusiform gyrus, cerebellum 6, vermis 1 and vermis 2, parahippocampus (PARAHIPP), superior parietal gyrus, middle frontal gyrus, inferior frontal gyrus (triangular part), middle temporal gyrus, calcarine fissure, precuneus, cuneus, inferior frontal gyrus (opercular part), superior occipital gyrus, precentral gyrus, and postcentral gyrus, and for min SHAM they were the inferior frontal gyrus (orbital part), inferior temporal gyrus, caudate, anterior cingulate, superior frontal gyrus, middle frontal gyrus, angular gyrus, precuneus, supramarginal gyrus, and inferior parietal gyrus.

Brain regions comparing activations between ACU versus min SHAM are indicated in Table 2. ROI used for the comparisons was the left AMY. This was an uncorrected comparison set at *P* = 0.005 with a *T* value of 2.898. The key regions for ACU were the inferior frontal gyrus (orbital part), temporal pole: superior temporal gyrus, middle frontal gyrus (orbital part), olfactory cortex, cerebellum 3 and cerebellum 6, inferior occipital gyrus, vermis 3, gyrus rectus, anterior cingulate, middle temporal gyrus, superior frontal gyrus (medial orbital part), superior frontal gyrus, and supraspinal nucleus, and for min SHAM they included the putamen, HIPP, lingual gyrus, inferior temporal gyrus, INS, inferior frontal gyrus (orbital part), PARAHIPP, temporal pole: superior temporal gyrus, fusiform gyrus, cerebellum 8, calcarine fissure, thalamus, heschl gyrus, superior frontal gyrus, middle frontal gyrus, middle occipital gyrus, superior occipital gyrus, angular gyrus, inferior parietal gyrus, superior parietal gyrus, precentral gyrus, and median cingulate.

Brain regions comparing activations between ACU versus min SHAM are shown in Table 3. ROI used for the comparisons was the right AMY. This was an uncorrected comparison set at *P* = 0.005 with a *T* value of 2.898. The key regions for ACU were the cerebellum 4-5, lingual gyrus, inferior frontal gyrus (orbital part), cerebellum 6, fusiform gyrus, inferior temporal gyrus, cerebellum crus 2, middle temporal gyrus, superior frontal gyrus, inferior frontal gyrus (opercular part), supramarginal gyrus, cuneus, and superior parietal gyrus, and for min SHAM they were the inferior temporal gyrus, cerebellum crus 2, INS, superior temporal gyrus, putamen, middle frontal gyrus, inferior frontal gyrus (opercular part), heschl gyrus, middle temporal gyrus, rolandic operculum, supramarginal gyrus, precentral gyrus, paracentral lobule, and postcentral gyrus.

Comparisons of common brain regions activated between ACU versus min SHAM are listed in Table 4. ROIs used for the comparisons were the HYP and the right and left AMY. These uncorrected comparisons were all set at *P* = 0.005 with a *T* value of 2.898. The middle frontal gyrus and precuneus were the overlapping regions for the HYP. The inferior frontal gyrus (orbital part), temporal pole: superior temporal gyrus, and superior frontal gyrus were the overlapping regions for the left AMY. The inferior temporal gyrus, cerebellum crus 2, middle temporal gyrus, inferior frontal gyrus (opercular part), and the supramarginal gyrus were the overlapping regions for the right AMY.

**Figure 4:**
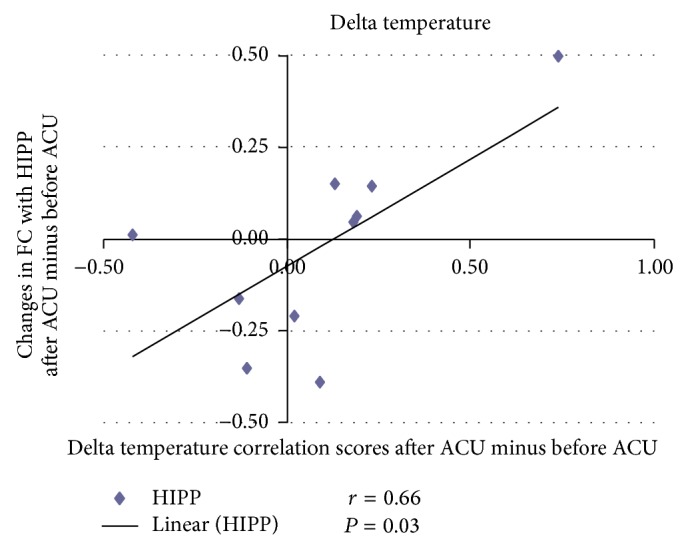
Changes in functional connectivity (FC) with the hippocampus (HIPP) after acupunture minus before acupuncture (ACU) in relation to delta temperature correlation scores after ACU minus before ACU. Increased hypothalamus-hippocampus functional connectivity (HYP–HIPP FC) after acupuncture (after ACU) was positively correlated with ACU-induced change in core body temperature suggesting that increased dopamine modulation during ACU was possibly associated with increased poststimulation limbic system and spinothalamic tract connectivity (*r* = 0.66; *P* = 0.03).

**Figure 5:**
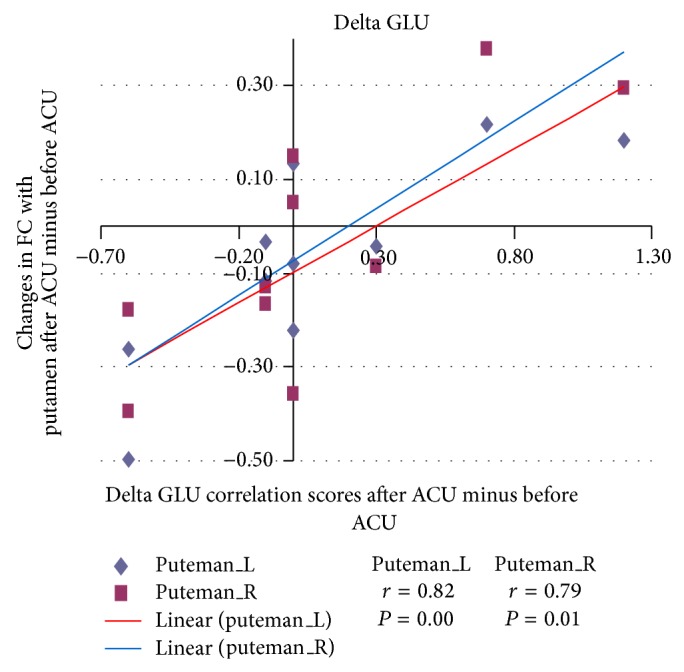
Changes in functional connectivity (FC) with the putamen (PUT) after acupuncture minus before acupuncture (ACU) in relation to delta glucose (GLU) correlation scores after ACU minus before ACU. Increased hypothalamus-putamen (L = left; R = right) functional connectivity (HYP-PUT FC) after acupuncture (after ACU) was positively correlated with ACU-induced change in GLU suggesting that increased dopamine modulation during ACU was probably associated with increased poststimulation limbic system and spinothalamic tract connectivity (left putamen *r* = 0.82, *P* = 0.00; right putamen *r* = 0.79, *P* = 0.01).

**Figure 6:**
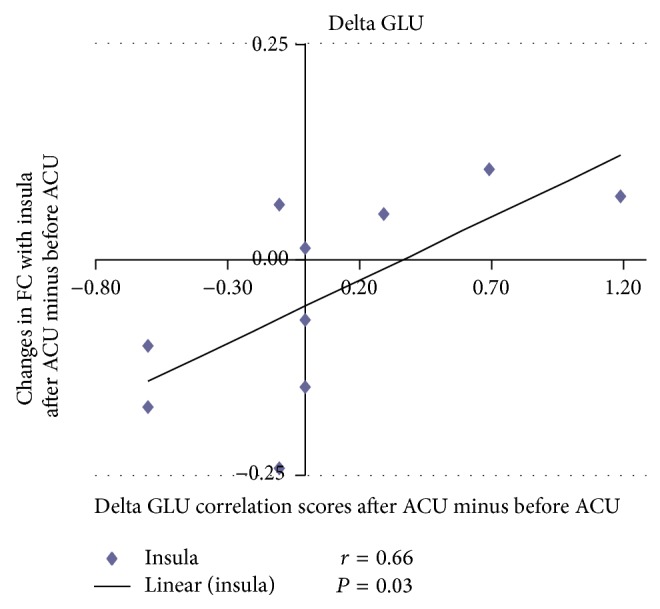
Changes in functional connectivity (FC) with the insula after acupuncture minus before acupuncture (ACU) in relation to delta glucose (GLU) correlation scores after ACU minus before ACU. Increased hypothalamus-insula functional connectivity (HYP-INS FC) after acupuncture (after ACU) was positively correlated with ACU-induced change in GLU suggesting that increased dopamine modulation during ACU was deemed to be associated with increased poststimulation limbic system and spinothalamic tract connectivity (*r* = 0.66; *P* = 0.03).

**Figure 7:**
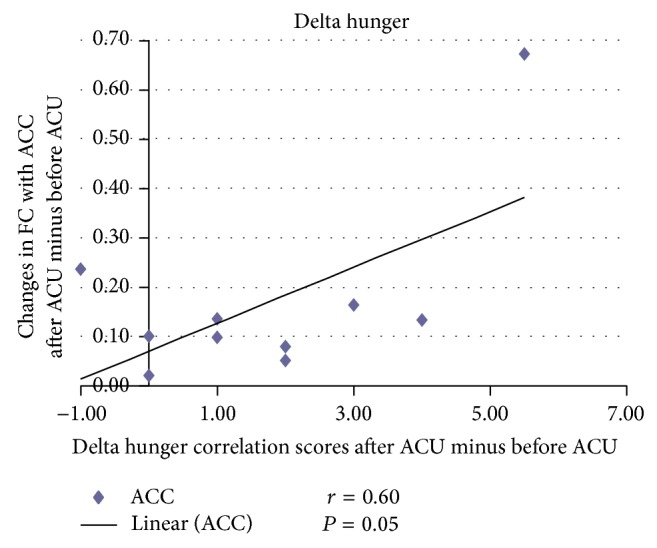
Changes in functional connectivity (FC) with the anterior cingulate cortex (ACC) after acupuncture minus before acupuncture (ACU) in relation to delta hunger correlation scores after ACU minus before ACU. Increased hypothalamus-ACC functional connectivity (HYP-ACC FC) after acupuncture (after ACU) was positively correlated with ACU-induced change in hunger suggesting that increased dopamine modulation during ACU was presumably associated with increased poststimulation limbic system and spinothalamic tract connectivity (*r* = 0.60; *P* = 0.05).

**Figure 8:**
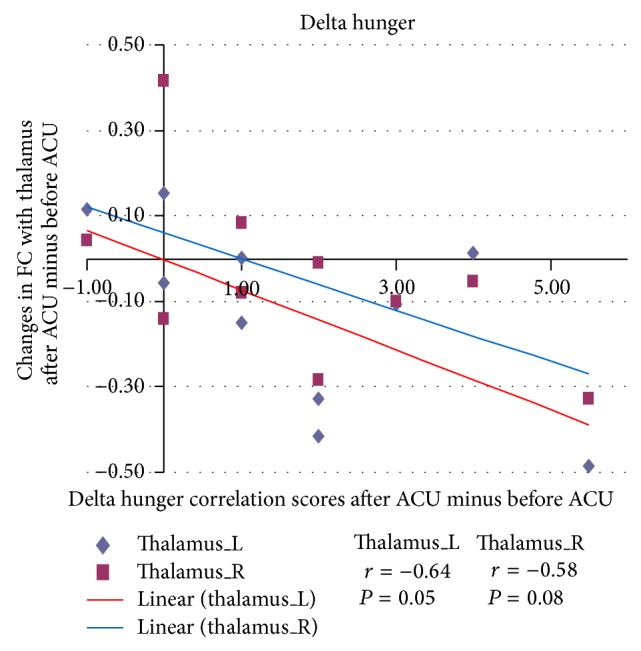
Changes in functional connectivity (FC) with the thalamus after minus before acupuncture (ACU) in relation to delta hunger correlation scores after ACU minus before ACU. Decreased hypothalamus-thalamus (L: left; R: right) functional connectivity (HYP-THALAMUS FC) after acupuncture (after ACU) was negatively correlated with ACU-induced change in hunger suggesting that increased dopamine modulation during ACU was most likely associated with decreased poststimulation limbic system and spinothalamic tract connectivity (left thalamus *r* = −0.64, *P* = 0.05; right thalamus *r* = −0.58, *P* = 0.08).

For the correlation analysis, normalized individual response to ACU and min SHAM was calculated by subtracting the value in the prestimulation rest from the value during active stimulation. To investigate the relationship between these metrics and imaging results (significant after ACU minus before ACU change in mean* z*-statistic within ROIs from the paired *t*-test above), Pearson correlation coefficients were calculated at a significance level of *P* < 0.05 (a lower *P* value could not be used to detect any regions). No correlation was found in min SHAM. ACU increased the *z*-scores of the differences in certain brain regions. A positive correlation coefficient meant that as the value of one variable increased, the value of the other variable increased; as one decreased the other decreased. A negative correlation coefficient indicated that as one variable increased, the other decreased and vice versa. Using our FC results, we used Pearson correlation to suggest causal relationships. As a result, increased HYP–HIPP FC after ACU was positively correlated with ACU-induced change in CBT suggesting that increased DA modulation during ACU was probably associated with increased poststimulation limbic system and spinothalamic tract connectivity, as shown in Figure 4. Increased HYP-PUT-INS FC after ACU was positively correlated with ACU-induced change in GLU suggesting that increased dopamine (DA) modulation during ACU was possibly associated with increased poststimulation limbic system and spinothalamic tract connectivity, as shown in Figures 5 and 6. Increased HYP-anterior cingulate cortex (ACC) FC after ACU was positively correlated with ACU-induced change in HUNGER suggesting that increased DA modulation during ACU was conceivably associated with increased poststimulation limbic system and spinothalamic tract connectivity, as shown in Figure 7. Decreased HYP-THALAMUS FC after ACU was negatively correlated or anticorrelated with ACU-induced change in HUNGER suggesting that increased DA modulation during ACU was perhaps associated with decreased poststimulation limbic system and spinothalamic tract connectivity, as shown in Figure 8. All we can deduce is that the two variables occurred together, so that changes in one were accompanied by systematic changes in the other. Causal inferences were made based on underlying theories and knowledge.

Based on the overall results above, it is reasonable to assume that ACU at the chosen acupoints modulated the spinothalamic tract and limbic system as well as areas of DA regulation. The brain areas that overlapped the treatment versus control group were involved in executive functions, default mode network, language, hearing and sensory information processing, go/no-go task regulation, cognition, emotion processing, and face recognition [22, 27–32]. Even though the AMY and HYP are linked, there were differences between their connectivities. For instance, when the HYP was the ROI for ACU, the main areas were assumed to be correlated with executive brain functions involving visual regulation, default mode network, face and body recognition, language centers, limbic system, emotion processing, satiety and hunger regulation, memory processing, primary motor cortex function, primary somatosensory function, go/no-go task regulation, thalamocortical input, glycometabolism regulation, and consciousness [7, 11, 20, 23, 27–32]. When the HYP was the ROI for min SHAM, the main areas seemed to be correlated with vision control, speech and language regulation, sensory information processing, executive brain functions, memory processing, and self-awareness [22, 27–32]. When the left AMY was the ROI for ACU, the main areas appeared to be correlated with self-awareness and sensory information processing including smell, face recognition, limbic system and DA production, speech and language centers, visual processing, glycometabolism regulation, and go/no-go task regulation [1, 20, 27–32]. When the left AMY was the ROI for min SHAM, the main areas gave the impression of being correlated with auditory and vision control, language and speech centers, homeostasis, DA production, cognitive and executive functions, memory regulation, face and body recognition, emotion and sensory processing, and CBT regulation [10, 22, 27–33]. When the right AMY was the ROI for ACU, the main areas were presumed to be correlated with cognition, emotion regulation, language centers, visual and hearing processing, face recognition, memory processing, and go/no-go task regulation [22]. Finally, when the right AMY was the ROI for min SHAM, the main areas came across as being correlated with face perception, DA regulation, homeostasis, primary motor cortex control, go/no-go task regulation, hearing and language regulation, and primary somatosensory center regulation [10, 27–33].

The AMY and HYP were chosen as ROIs for the simple reason that the various regions of the HYP are involved in appetite control and thermoregulation. The AMY is often linked with the HYP [34]. As shown in Figures 2 and 3, there was a significant difference in the spatial patterns of the distinct brain regions between the two treatment groups as well as between the ROIs. As defined previously, functional connectivity describes the temporal synchrony or correlation of the BOLD fMRI signal from two or more anatomically separated brain regions [25]. Therefore, the spatial and temporal patterns of brain responses could be modulated by the sustained effects of ACU versus min SHAM. We derived the functional connectivity networks from the temporal pattern of the states during and after stimulation associated with the ROIs and the interconnected regions. This is along the lines of our hypotheses that the mPFC, cerebral cortex, cerebellum, DLPFC, VMPFC, and others would be activated due to the acute ACU effects affecting primarily satiety, metabolism, and some cognitive functions. With respect to the connectivity analyses, primary somatosensory, motor function, visual stimulation, language, limbic system (pain), and cognitive function centers were determined to be involved in both ACU and min SHAM. This was expected since the sensation of the needle and the surrounding environment stimulated all subjects' brain areas as noted in the limbic structures that are discussed further.

Previous human neuroimaging studies have shown that acupuncture stimulation activates extensive brain regions, including the primary somatosensory cortex (SI), secondary somatosensory cortex (SII), ACC, insular cortex, PFC, AMY, HIPP, periaquaductal gray (PAG), and HYP [27–32]. These distributed brain regions are closely associated with a wider pain matrix for modulating sensations and affective pain perception especially pertaining to* De-qi* or acupuncture sensations. Acute pain from superficial needling has been linked with deactivations located in the fusiform gyrus, PARAHIPP, and posterior temporal lobe. For deep needling, the deactivations were located in the posterior temporal lobe, cerebellum, and thalamus [35].

Based on the connectivity results with the AMY and HYP, it can be assumed that the mode of action for ACU and min SHAM is mediated by the limbic system, specifically the neurotransmitter dopamine (DA). DA is known to increase heart rate and blood pressure [33]; hence it would affect core body temperature in our subjects as shown by the activation of the supraspinal nucleus. DA also has a role in pain processing [36], which would explain* De-qi* or sensations felt during ACU stimulation. This conclusion is based on ACU activation of the supraspinal nucleus (autonomic function in circulation and respiratory regulation), ACC, HYP, and HIPP [33, 36]. DA also has site-specific action regulating the intake of food; it reinforces the effects of food [37]. DA is necessary to begin the meal process [38]. It acts upon the prefornical area, VMH, and ARC to reduce the consumption of food and prevent hyperphagia, which in turn is affected by leptin, insulin, and other hormones [39]. It may be inferred that disruptions in DA production and/or structure may predispose certain individuals to obesity.

One important observation was that, with activations in the AMY, ACC, DLPFC, inferior temporal gyrus, and PARAHIPP, the subjects were probably thinking about food,* De-qi* sensations, and/or hunger. Therefore, there was strong evidence to support a feasible direct correlation between behavioral data and the functional connectivity results [40]. For example, the ACC is of great importance in this study since it is involved in blood pressure regulation and heart rate, but it also shares direct connection with the AMY, HYP, nACC, and INS [41]. Specifically, activity of the right anterior insula was correlated with hunger whereas activity of the right posterior INS was correlated with satiety [42]. The ARC and VMH may even be activated centrally to uptake GLU [20] via the effects of ACU. As for the physiological data [40], it can be inferred that the reason for the variability amongst treatment groups was due to the fact that ACU is tailored to the unique physiology of each individual despite having a homogeneous experimental population.

Another important point of consideration was acupoint specificity. In a study by Hui et al. (2005), stimulation at ST 36 led to signal decreases in various limbic/subcortical structures [31]. Wu and colleagues further suggested that higher behavioral scores associated with* De-qi* sensations during acupuncture at LI 4 and ST 36 were linked to the deactivation of multiple limbic system structures [21]. Compared with acupuncture at a nonacupoint, acupuncture at ST 36 caused more complex response patterns with more vast spatial distributions such as intermittent activity in brainstem structures and HYP [43], as reflected in our results. Overall, it would be best to test a variety of acupoints or a combination thereof relating to the desired physiological outcomes and acupoint specificity in future studies.

Limitations in most acupuncture studies as well as in our previous study [40] must have subjects that meet the inclusion criteria and have adequate sample size. Although the current study's subject number size was considered to be small, there are numerous studies [44–47] in acupuncture that provided valid results with 20 subjects or less such as in our pilot study [40]. Most importantly, using single gender subjects is also widely accepted in the literature [48] to avoid gender differences and to enhance subject homogeneity, particularly in functional imaging studies. Therefore, the emphasis should be on the fMRI aspect and findings in this study. To obtain repeatable and valid results with the fewest confounding factors, a representative homogeneous male population in a specific age bracket (young adults only) was chosen. Choosing women in that age group for this pilot study may have confounded the results with hormonal interplay and variability. The current researchers plan to include females, normal, and obese individuals in a future larger study. Other confounding factors included* De-qi* mixed with pain, artifactual activation, appropriate controls, patient anxiety, selection of appropriate type and number of acupoints to obtain adequate results, and anticipation of pain or discomfort from acupuncture treatment [40]. In this particular study [40], it was difficult to obtain an ideal overweight population. In the Sichuan Province (China), the BMI of the subjects was much lower than in other countries or regions, but the subjects had a higher WHR (>0.9), which led the current research team to classify the subjects as being “overweight” or “unfit” in principle. Most importantly, a 2007 epidemiological survey conducted in the Sichuan Province for people above the age of 18 classified these individuals as overweight at a lower BMI and thus supported the selection of the BMI range in this pilot study (>18 to <30 BMI) [49]. This could be a result of dietary (hot, spicy food) and lifestyle (genetic hypertension and smoking) factors [49].

## 4. Conclusions

This study is of importance in depicting possible mechanisms for the neurophysiological results (not shown) from our previous study [40]. There were significant differences as well as few similarities (*P* < 0.05) in the connectivity results between ACU and min SHAM based on our results. Most importantly, our data supported the finding of a possible direct correlation between behavioral data and the functional connectivity results and reinforced findings from previous acupuncture experiments [27–32]. Future studies are needed to expand on the theoretical mechanisms of acupuncture in overweight and obese populations.

## Figures and Tables

**Table 1:** Brain regions comparing activations between real acupuncture (ACU) versus minimal sham acupuncture (SHAM). Activations indicate whether ACU was greater than SHAM or SHAM was greater than ACU. Region of interest (ROI) used for the comparisons was the HYP. This was an uncorrected comparison set at *P* = 0.005 with a *T* value of 2.898.

Brain areas	Activation	Side	*P* value	*T* value
STG	ACU > SHAM	R	0.005	2.898
MOG	ACU > SHAM	L	0.005	2.898
Vermis 3	ACU > SHAM	NA	0.005	2.898
TPOsup	ACU > SHAM	R	0.005	2.898
HIPP	ACU > SHAM	B	0.005	2.898
FFG	ACU > SHAM	L	0.005	2.898
Cerebellum 6	ACU > SHAM	B	0.005	2.898
Vermis 1 and vermis 2	ACU > SHAM	NA	0.005	2.898
PARAHIPP	ACU > SHAM	R	0.005	2.898
SPG	ACU > SHAM	L	0.005	2.898
MFG	ACU > SHAM	R	0.005	2.898
IFGtriang	ACU > SHAM	B	0.005	2.898
MTG	ACU > SHAM	B	0.005	2.898
CAL	ACU > SHAM	R	0.005	2.898
PCUN	ACU > SHAM	R	0.005	2.898
CUN	ACU > SHAM	R	0.005	2.898
IFGoperc	ACU > SHAM	R	0.005	2.898
SOG	ACU > SHAM	L	0.005	2.898
PreCG	ACU > SHAM	R	0.005	2.898
PoCG	ACU > SHAM	L	0.005	2.898
ORBinf	SHAM > ACU	L	0.005	2.898
ITG	SHAM > ACU	L	0.005	2.898
CAU	SHAM > ACU	L	0.005	2.898
ACG	SHAM > ACU	L	0.005	2.898
SFG	SHAM > ACU	B	0.005	2.898
MFG	SHAM > ACU	B	0.005	2.898
ANG	SHAM > ACU	L	0.005	2.898
PCUN	SHAM > ACU	L	0.005	2.898
SMG	SHAM > ACU	L	0.005	2.898
IPL	SHAM > ACU	L	0.005	2.898

STG: superior temporal gyrus; MOG: middle occipital gyrus; TPOsup: temporal pole: superior temporal gyrus; HIPP: hippocampus; FFG: fusiform gyrus; PARAHIPP: parahippocampus; SPG: superior parietal gyrus; MFG: middle frontal gyrus; IFGtriang: inferior frontal gyrus (triangular part); MTG: middle temporal gyrus; CAL: calcarine fissure; PCUN: precuneus; CUN: cuneus; IFGoperc: inferior frontal gyrus (opercular part); SOG: superior occipital gyrus; PreCG: precentral gyrus; PoCG: postcentral gyrus; ORBinf: inferior frontal gyrus (orbital part); ITG: inferior temporal gyrus; CAU: caudate nucleus; ACG: anterior cingulate; SFG: superior frontal gyrus; ANG: angular gyrus; SMG: supramarginal gyrus; IPL: inferior parietal gyrus; NA: not applicable; L: left; B: bilateral; R: right.

**Table 2:** Brain regions comparing activations between real acupuncture (ACU) versus minimal sham acupuncture (SHAM). Activations indicate whether ACU was greater than SHAM or SHAM was greater than ACU. Region of interest (ROI) used for the comparisons was the left amygdala. This was an uncorrected comparison set at *P* = 0.005 with a *T* value of 2.898.

Brain areas	Activation	Side	*P* value	*T* value
ORBinf	ACU > SHAM	B	0.005	2.898
TPOsup	ACU > SHAM	B	0.005	2.898
ORBmid	ACU > SHAM	R	0.005	2.898
OLF	ACU > SHAM	L	0.005	2.898
Cerebellum 3 and cerebellum 6	ACU > SHAM	R	0.005	2.898
IOG	ACU > SHAM	R	0.005	2.898
Vermis 3	ACU > SHAM	NA	0.005	2.898
REC	ACU > SHAM	B	0.005	2.898
ACG	ACU > SHAM	B	0.005	2.898
MTG	ACU > SHAM	B	0.005	2.898
ORBsupmed	ACU > SHAM	B	0.005	2.898
SFG	ACU > SHAM	R	0.005	2.898
Supraspinal nucleus	ACU > SHAM	L	0.005	2.898
PUT	SHAM > ACU	R	0.005	2.898
HIPP	SHAM > ACU	R	0.005	2.898
LING	SHAM > ACU	L	0.005	2.898
ITG	SHAM > ACU	B	0.005	2.898
INS	SHAM > ACU	R	0.005	2.898
ORBinf	SHAM > ACU	R	0.005	2.898
PARAHIPP	SHAM > ACU	B	0.005	2.898
TPOsup	SHAM > ACU	L	0.005	2.898
FFG	SHAM > ACU	R	0.005	2.898
Cerebellum 8	SHAM > ACU	B	0.005	2.898
CAL	SHAM > ACU	L	0.005	2.898
THA	SHAM > ACU	L	0.005	2.898
HES	SHAM > ACU	L	0.005	2.898
SFG	SHAM > ACU	R	0.005	2.898
MFG	SHAM > ACU	B	0.005	2.898
MOG	SHAM > ACU	R	0.005	2.898
SOG	SHAM > ACU	R	0.005	2.898
ANG	SHAM > ACU	B	0.005	2.898
IPL	SHAM > ACU	L	0.005	2.898
SPG	SHAM > ACU	R	0.005	2.898
PreCG	SHAM > ACU	B	0.005	2.898
DCG	SHAM > ACU	L	0.005	2.898

ORBinf: inferior frontal gyrus (orbital part); TPOsup: temporal pole: superior temporal gyrus; ORBmid: middle frontal gyrus (orbital part); OLF: olfactory cortex; IOG: inferior occipital gyrus; REC: gyrus rectus; ACG: anterior cingulate; MTG: middle temporal gyrus; ORBsupmed: superior frontal gyrus (medial orbital part); SFG: superior frontal gyrus; PUT: putamen; HIPP: hippocampus; LING: lingual gyrus; ITG: inferior temporal gyrus; INS: insula; PARAHIPP: parahippocampus; FFG: fusiform gyrus; CAL: calcarine fissure, THA: thalamus; HES: heschl gyrus; MFG: middle frontal gyrus; MOG: middle occipital gyrus; SOG: superior occipital gyrus; ANG: angular gyrus; IPL: inferior parietal gyrus; SPG: superior parietal gyrus; PreCG: precentral gyrus; DCG: median cingulate; NA: not applicable; L: left; B: bilateral; R: right.

**Table 3:** Brain regions comparing activations between real acupuncture (ACU) versus minimal sham acupuncture (SHAM). Activations indicate whether ACU was greater than SHAM or SHAM was greater than ACU. Region of interest (ROI) used for the comparisons was the right amygdala. This was an uncorrected comparison set at *P* = 0.005 with a *T* value of 2.898.

Brain areas	Activation	Side	*P* value	*T* value
Cerebellum 4 and cerebellum 5	ACU > SHAM	L	0.005	2.898
LING	ACU > SHAM	B	0.005	2.898
ORBinf	ACU > SHAM	L	0.005	2.898
Cerebellum 6	ACU > SHAM	B	0.005	2.898
FFG	ACU > SHAM	L	0.005	2.898
ITG	ACU > SHAM	L	0.005	2.898
Cerebellum crus 2	ACU > SHAM	L	0.005	2.898
MTG	ACU > SHAM	R	0.005	2.898
SFG	ACU > SHAM	R	0.005	2.898
IFGoperc	ACU > SHAM	R	0.005	2.898
SMG	ACU > SHAM	R	0.005	2.898
CUN	ACU > SHAM	R	0.005	2.898
SPG	ACU > SHAM	L	0.005	2.898
ITG	SHAM > ACU	L	0.005	2.898
Cerebellum crus 2	SHAM > ACU	L	0.005	2.898
INS	SHAM > ACU	L	0.005	2.898
STG	SHAM > ACU	R	0.005	2.898
PUT	SHAM > ACU	B	0.005	2.898
MFG	SHAM > ACU	B	0.005	2.898
IFGoperc	SHAM > ACU	L	0.005	2.898
HES	SHAM > ACU	R	0.005	2.898
MTG	SHAM > ACU	R	0.005	2.898
ROL	SHAM > ACU	L	0.005	2.898
SMG	SHAM > ACU	R	0.005	2.898
PreCG	SHAM > ACU	B	0.005	2.898
PCL	SHAM > ACU	L	0.005	2.898
PoCG	SHAM > ACU	R	0.005	2.898

LING: lingual gyrus; ORBinf: inferior frontal gyrus (orbital part); FFG: fusiform gyrus; ITG: inferior temporal gyrus; MTG: middle temporal gyrus; SFG: superior frontal gyrus; IFGoperc: inferior frontal gyrus (opercular part); SMG: supramarginal gyrus; CUN: cuneus; SPG: superior parietal gyrus; ITG: inferior temporal gyrus; INS: insula; STG: superior temporal gyrus; PUT: putamen; MFG: medial frontal gyrus; HES: heschl gyrus; ROL: rolandic operculum; PreCG: precentral gyrus; PCL: paracentral lobule; PoCG: postcentral gyrus; L: left; B: bilateral; R: right.

**Table 4:** Comparison of common brain regions activated between real acupuncture (ACU) versus minimal sham acupuncture (SHAM). Regions of interest (ROIs) used for the comparisons were the hypothalamus (HYP) and left and right amygdala (AMY). These uncorrected comparisons were set at *P* = 0.005 with a *T* value of 2.898.

Region of interest	Common brain regions	*P* value	*T* value
HYP	MFG and PCUN	0.005	2.898
LEFT AMY	ORBinf, TPOsup, and SFG	0.005	2.989
RIGHT AMY	ITG, cerebellum crus 2, MTG, IFGoperc, and SMG	0.005	2.898

MFG: medial frontal gyrus; PCUN: precuneus; ORBinf: inferior frontal gyrus (orbital part); TPOsup: temporal pole: superior temporal gyrus; SFG: superior frontal gyrus; ITG: inferior temporal gyrus; MTG: middle temporal gyrus; IFGoperc: inferior frontal gyrus (opercular part); SMG: supramarginal gyrus.
